# Targeted Multidisciplinary Continuous Improvement Process Reduces Time to Breast Cancer Surgery with Immediate Reconstruction

**DOI:** 10.1245/s10434-026-20143-z

**Published:** 2026-07-14

**Authors:** Pooja M. Varman, Rahul Rangan, Amanda Maggiotto, Vincent Wu, Risal Djohan, Zahraa Al-Hilli

**Affiliations:** 1 Department of General Surgery, Digestive Disease Institute, Cleveland Clinic, Cleveland, OH USA; 2 Breast Center, Integrated Surgical Institute, Cleveland Clinic, Cleveland, OH USA; 3 Taussig Cancer Institute, Cleveland Clinic, Cleveland, OH USA; 4 Department of Plastic Surgery, Integrated Surgical Institute, Cleveland, OH USA

## Abstract

**Background:**

Breast surgery with immediate breast reconstruction (IBR) is associated with delays in definitive surgery and adjuvant therapy. We evaluated a multiphase quality improvement (QI) initiative to reduce time to treatment (TTT) for patients undergoing upfront surgery with IBR.

**Methods:**

This retrospective cohort study included patients with newly diagnosed breast cancer undergoing upfront surgery at a single institution between January 2022 and June 2025. Patients with metastatic disease, neoadjuvant therapy, or first treatment outside this institution were excluded. The QI initiative comprised three phases: (1) weekly multidisciplinary team huddle; (2) standardized communication protocol; and (3) OR alignment between breast and plastic surgery. Primary outcome was median TTT. Time to treatment for non-IBR patients was tracked concurrently as a balancing metric.

**Results:**

A total of 3,324 patients met inclusion criteria; 1,100 (33.1%) underwent surgery with IBR and 2,224 (66.9%) without. The cohort was predominantly early-stage (82.8% cTis/T1). Median TTT declined from 56 to 36 days in the IBR group and from 41 to 29 days in the non-IBR group (both *P* < 0.001). The proportion waiting longer than 45 days declined from 67.4 to 27.3% for IBR and from 40.5 to 22.9% for non-IBR (both *P* < 0.001). Only the IBR group demonstrated statistically significant improvement through Phase 3 (*P* < 0.001), while the non-IBR group plateaued after Phase 2.

**Conclusions:**

A targeted multidisciplinary QI initiative meaningfully reduced TTT for breast cancer patients undergoing upfront surgery with IBR without delaying non-IBR patients. Operating room (OR) alignment was the pivotal intervention, highlighting the importance of system-level coordination between breast and plastic surgery.

For patients with early-stage breast cancer, upfront surgical resection is the cornerstone of treatment, and timeliness is a critical dimension of care. Delays from diagnosis to surgery have been associated with decreased overall and disease-specific survival across stages I and II, with breast cancer–specific mortality increasing with each successive interval of delay.^1^ In recognition of this, the Commission on Cancer (CoC) recommends that for patients with AJCC clinical stage I–III breast cancer, the first therapeutic surgery in a nonneoadjuvant setting be performed within 60 days of diagnosis.^2^

Immediate breast reconstruction (IBR) following mastectomy in the United States is on the rise, with more patients opting for IBR each year.^3^ Immediate breast reconstruction offers benefits in psychosocial wellbeing, body image, and quality of life. Patients who undergo IBR report significantly higher scores on validated measures of psychosocial, sexual, and physical wellbeing compared to those undergoing mastectomy without reconstruction.^4^ Despite its well-described benefits, IBR also introduces logistical complexity into the surgical care pathway. Unlike oncologic resection alone, IBR requires coordinated operative scheduling between two independent surgical services—breast surgical oncology and plastic and reconstructive surgery—each with distinct preoperative workup requirements, separate clinic workflows, and sometimes competing operative availability. This dependency on coordination between two surgical teams creates a structural bottleneck that may delay time to surgery by up to 2 weeks compared with nonreconstructive breast surgery, delaying not only definitive cancer surgery but also the initiation of adjuvant therapy.^5–7^

Early referral to plastic surgery and surgeon flexibility in operative scheduling have been identified as modifiable factors associated with reduced TTT in the IBR population.^8^ However, institution-level, multiphase quality improvement (QI) interventions that systematically address the structural barriers specific to IBR scheduling and rigorously evaluate their impact on TTT remain limited in the literature. The purpose of this study was to evaluate the effectiveness of a multiphase QI initiative in reducing TTT for patients with breast cancer undergoing upfront surgery with IBR.

## Methods

### Study Population

This was a retrospective cohort study conducted at a single quaternary academic medical center across six practice sites. The study was approved by the Cleveland Clinic Institutional Review Board. We identified all patients with newly diagnosed breast cancer who underwent upfront surgery at our institution between January 2022 and September 2025. Eligible primary breast procedures included breast-conserving surgery (BCS) without IBR, BCS with IBR, mastectomy without IBR, and mastectomy with IBR. Immediate breast reconstruction procedures encompassed oncoplastic breast reduction, implant-based reconstruction (tissue expander or direct-to-implant), autologous tissue reconstruction (including pedicled and free flap techniques). Patients were excluded if they had metastatic disease, received neoadjuvant systemic therapy prior to surgery, or if their surgery was performed at an outside institution.

All eligible patients were stratified into two cohorts: (1) surgery with IBR, and (2) surgery without IBR. The non-IBR cohort served as a balancing metric to evaluate whether scheduling improvements for IBR patients had an adverse effect on wait time for patients undergoing breast surgery without reconstruction.^9^ Data were abstracted from an institutional dashboard, tumor registry, and chart review. Variables included patient demographics (age at diagnosis, sex, race and ethnicity), institution of diagnosis (same institution vs. outside referral), tumor characteristics (clinical T and N stage), date of diagnostic biopsy, dates of breast and plastic surgery consultations, date of initial surgical treatment, and procedure details, including type of breast procedure and reconstruction modality.

### The QI Initiative

A structured continuous improvement (CI) initiative using Lean methodology to optimize the time to treatment process was conducted. This first started with current-state process mapping across the patient pathway to identify workflow inefficiencies, delays, and barriers to care. Root cause analysis was undertaken using standard CI tools to characterize the barriers to timely care. Identified drivers were then prioritized through impact-effort assessment, and countermeasures were ranked accordingly. The top three were selected as high-impact interventions and were used in the process improvement initiative.

1. Baseline (January 2022–September 2022)
2. Phase 1 (October 2022–August 2023): Establishment of a weekly huddle to facilitate real-time identification of barriers to time to surgery and address delays. The weekly multidisciplinary huddle included all providers throughout the care delivery process; for example, breast and plastic surgeons, medical and radiation oncologists, nurses, advanced practice providers, schedulers and administrators. At each huddle, all patients who had been evaluated by a breast surgeon and were awaiting surgery were reviewed systematically. Particular attention was paid to patients with outstanding preoperative workup, unscheduled cases, or surgery dates over a 45-day threshold. For these patients, care was actively expedited by prioritizing pending imaging, earlier plastic surgery consultation, and/or direct scheduling intervention by the team.
3. Phase 2 (September 2023–January 2025): A standardized communication pathway was established through a designated point of contact within the Plastic Surgery Department to facilitate coordination of breast and plastics surgery cases. When a breast surgeon identified a patient requiring IBR, the point of contact was notified, who then reviewed all plastic surgeons’ schedules to identify the earliest available coordinated OR date.
4. Phase 3 (February 2025–September 2025): Alignment of operating room time between breast and plastic surgeons and identification of designated days for combined case scheduling based on individual breast surgeon needs assessment. Operative alignment was designed by using an internally data-driven approach rather than a fixed block time model. We began by analyzing the volume of IBR cases performed by each breast surgeon per month to determine the number of coordinated OR days required across the program. We then mapped each breast surgeon's existing OR days against those of each plastic surgeon to identify scheduling overlap. Based on the intersection of case volume needs and surgeon availability, we allocated specific coordinated OR days for defined breast surgeon–plastic surgeon pairings.

### Outcome Measures

The primary outcome was median TTT, defined as the number of days from the date of diagnostic biopsy to the date of initial surgical treatment. Secondary outcomes included the absolute phase-to-phase reduction in median TTT within each cohort and the proportion of patients in each cohort with TTT exceeding 45 days (an internal institutional metric for identifying outliers experiencing delays in treatment). The balancing metric, median TTT for the non-IBR cohort, was tracked concurrently across all phases.

### Statistical Analysis

Continuous variables are reported as median with interquartile range (IQR) and categorical variables as frequency and percentage. Between-phase TTT comparisons were performed using the Kruskal-Wallis test with post-hoc pairwise comparisons adjusted for multiple testing using the Bonferroni correction. Proportions of patients with TTT exceeding 45 days were compared across phases using the chi-square test. All tests were two-sided, and a *P* value less than 0.05 was considered statistically significant. This was a quality improvement study utilizing all eligible patients treated during the defined study period; therefore, no formal a priori sample size calculation was performed.

## Results

### Patient, Tumor, and Treatment Characteristics

A total of 3,324 patients with newly diagnosed breast cancer met inclusion criteria and underwent upfront surgery at our institution between January 2022 and June 2025. Median age at diagnosis was 64 years (IQR 54.5–73.5). Most of the cohort was female (*n* = 3,300, 99.3%). The cohort was 82.8% (*n* = 2,752) White, 9.4% (*n* = 312) Black, 2.4% (*n* = 81) Hispanic or Latino, 2% (*n* = 67) Asian, and 3.4% (*n* = 112) Other or Not Specified. Diagnosis was established at the same institution in 79.3% (*n* = 2,636) of patients and at an outside institution in 20.7% (*n* = 688). Clinical tumor stage distribution was Tis (*n* = 574, 17.3%), T1 (*n* = 2,183, 65.7%), T2 (*n* = 509, 15.3%), T3 (*n* = 52, 1.6%), and T4 (*n* = 7, 0.2%). N0 (*n* = 3,166, 95.2%), N1 (*n* = 154, 4.6%), and N2 (*n* = 4, 0.1%). Initial procedure was breast surgery with IBR in 33.1% (*n* = 1,100) of patients and breast surgery without IBR in 66.9% (*n* = 2,224). Primary oncologic procedures were BCS for 65.4% (*n* = 2,175) of patients and mastectomy for 34.6% (*n* = 1,149). IBR procedures were oncoplastic procedure or reduction (*n* = 387, 11.6%), implant-based reconstruction (*n* = 703, 21.1%), and autologous reconstruction (*n* = 10, 0.3%). Patient, tumor, and treatment characteristics are presented by cohort in Table 1.

**Table 1:** Demographic, tumor, and treatment characteristics

Demographic, tumor, and treatment characteristics	All patients *N* = 3,324 (100%)	With IBR *n* = 1,100 (33.1%)	Without IBR *n* = 2,224 (66.9%)	*P*
Median age at diagnosis [IQR]	64 [54.5–73.5]	55 [46.5–63.5]	67 [59.5–74.5]	**<0.001**
Female	3,300 (99.3)	1,100 (100)	2,200 (98.9)	**<0.001**
Race and ethnicity				**0.068**
White	2,752 (82.8)	900 (81.7)	1,852 (83.3)	
Black or African American	312 (9.4)	94 (8.5)	218 (9.8)	
Hispanic or Latino	81 (2.4)	36 (3.3)	45 (2.0)	
Asian	67 (2.0)	25 (2.3)	42 (1.9)	
Other or not specified	112 (3.4)	46 (4.2)	66 (3.0)	
Diagnosis location				**<0.001**
Internal	2,636 (79.3)	803 (73.0)	1,833 (82.4)	
External	688 (20.7)	297 (27.0)	391 (17.6)	
Clinical T				**<0.001**
cT0/is	574 (17.3)	267 (24.3)	307 (13.8)	
cT1	2,183 (65.7)	570 (51.8)	1,612 (72.5)	
cT2	509 (15.3)	231 (21.0)	278 (12.5)	
cT3	52 (1.6)	32 (2.9)	20 (0.9)	
cT4	7 (0.2)	0 (0)	7 (0.3)	
Clinical N				**0.046**
cN0	3,166 (94.2)	1,033 (93.9)	2,133 (95.9)	
cN1	154 (4.6)	65 (5.9)	89 (4.0)	
cN2	4 (0.1)	2 (0.2)	2 (0.1)	
Oncologic breast procedure				**<0.001**
Lumpectomy	2,175 (65.4)	378 (34.4)	1,797 (80.8)	
Mastectomy	1,149 (34.6)	722 (65.6)	427 (19.2)	
Reconstructive procedure				–
Oncoplastic procedure or reduction	387 (11.6)	382 (35.2)	–	
Implant-based reconstruction	703 (21.1)	703 (63.9)	–	
Autologous reconstruction	10 (0.3)	10 (0.9)	–	

Significant values (*P* < 0.05) shown in bold

*IBR* immediate breast reconstruction; *IQR* interquartile range

### Overall Impact of the QI Initiative

Across the full study period, median TTT improved in both cohorts with stepwise implementation of the QI initiative, declining from 46 days (IQR 34–62) overall at baseline to 32 days (IQR 23–44) in Phase 3 (*P* < 0.001). The proportion of patients waiting longer than 45 days for surgery also declined significantly from 50.3 to 24.2% (*P* < 0.001). Both the surgery with and without IBR groups demonstrated significant improvement over time; however, the magnitude and phase-to-phase patterns of improvement differed meaningfully between the two groups, as detailed below and summarized in Table 2.

**Table 2:** Phase-by-phase quality improvement outcomes, including median time to treatment and proportion of patients waiting longer than 45 days, by cohort

	With IBR				Without IBR			
	Median TTT, days (IQR)	*P*^a^	% >45 days	*P*^b^	Median TTT, days (IQR)	*P*^a^	% >45 days	*P*^b^
Baseline	56 (41–70)	–	67.4%	–	41 (30–55)	–	40.5%	–
Phase 1	51 (40–67)	0.590	63.3%	1.000	36 (27–48)	**0.002**	30%	**0.006**
Phase 2	43 (34–59)	**<0.001**	44.7%	**<.001**	31 (22–44)	**<0.001**	23%	**0.019**
Phase 3	36 (27–47)	**<0.001**	27.3%	**0.004**	29 (21–41)	0.332	22.9%	1.000
Baseline versus Phase 3 (overall)	56 versus 36	**<0.001**	67.4% versus 27.3%	**<0.001**	41 versus 29	**<0.001**	40.5% versus 22.9%	**<0.001**

*P* values are versus prior phase (consecutive), except the summary row (Phase 0 vs. Phase 3). Significant values (*P* < 0.05) shown in bold

^a^Bonferroni-corrected Mann-Whitney *U* test (two-sided) for median TTT. Adjusted *p* = raw p × 6, capped at 1.0

^b^Bonferroni-corrected chi-square test for proportion of patients waiting >45 days. Adjusted *p* = raw *p* × 6, capped at 1.0.

*IBR* immediate breast reconstruction; *IQR* interquartile range; *TTT* time to treatment (date of diagnostic biopsy to date of first surgical treatment)

### Surgery with IBR Cohort

In the surgery with IBR cohort, median TTT decreased from 56 days (IQR 41–70) in the pre-intervention period to 51 days (IQR 40–67) in Phase 1, 43 days (IQR 34–59) in Phase 2, and 36 days (IQR 27–47) in Phase 3 (*P* < 0.001 overall). The proportion of patients waiting longer than 45 days for surgery declined from 67.4% at baseline to 63.3% in Phase 1, 44.7% in Phase 2, and 27.3% in Phase 3 (*P* < 0.001 overall). Phase-to-phase pairwise comparisons after Bonferroni correction showed that the reduction from pre-intervention to Phase 1 did not reach statistical significance after Bonferroni correction (5-day reduction, adjusted *P* = 0.59). Significant reductions were observed from Phase 1 to Phase 2 (8-day reduction, adjusted *P* < 0.001) and from Phase 2 to Phase 3 (7 day reduction, adjusted *P* < 0.001). Median time between consultation appointment with surgical oncologist and plastic surgeon was 11 days (IQR 4–21), with no phase-to-phase difference observed.

### Surgery Without IBR Cohort

In the surgery without IBR cohort, median TTT decreased from 41 days (IQR 30–55) in the pre-intervention period to 36 days (IQR 27–48) in Phase 1, 31 days (IQR 22–44) in Phase 2, and 29 days (IQR 21–41) in Phase 3 (*P* < 0.001 overall). The proportion of patients waiting longer than 45 days for surgery declined from 40.5% at baseline to 30% in Phase 1, 23% in Phase 2, and 22.9% in Phase 3 (*P* < 0.001 overall). Phase-to-phase pairwise Bonferroni-corrected analysis demonstrated statistically significant reductions from pre-intervention to Phase 1 (5-day reduction, adjusted *P* = 0.002), and from Phase 1 to Phase 2 (5-day reduction, adjusted *P* < 0.001). However, the reduction from Phase 2 to Phase 3 was not statistically significant (2 days reduction, adjusted *P* = 0.332), indicating a plateau in improvement after Phase 2 in this cohort.

### Phase-to-Phase Divergence Between Cohorts

**Fig. 1:**
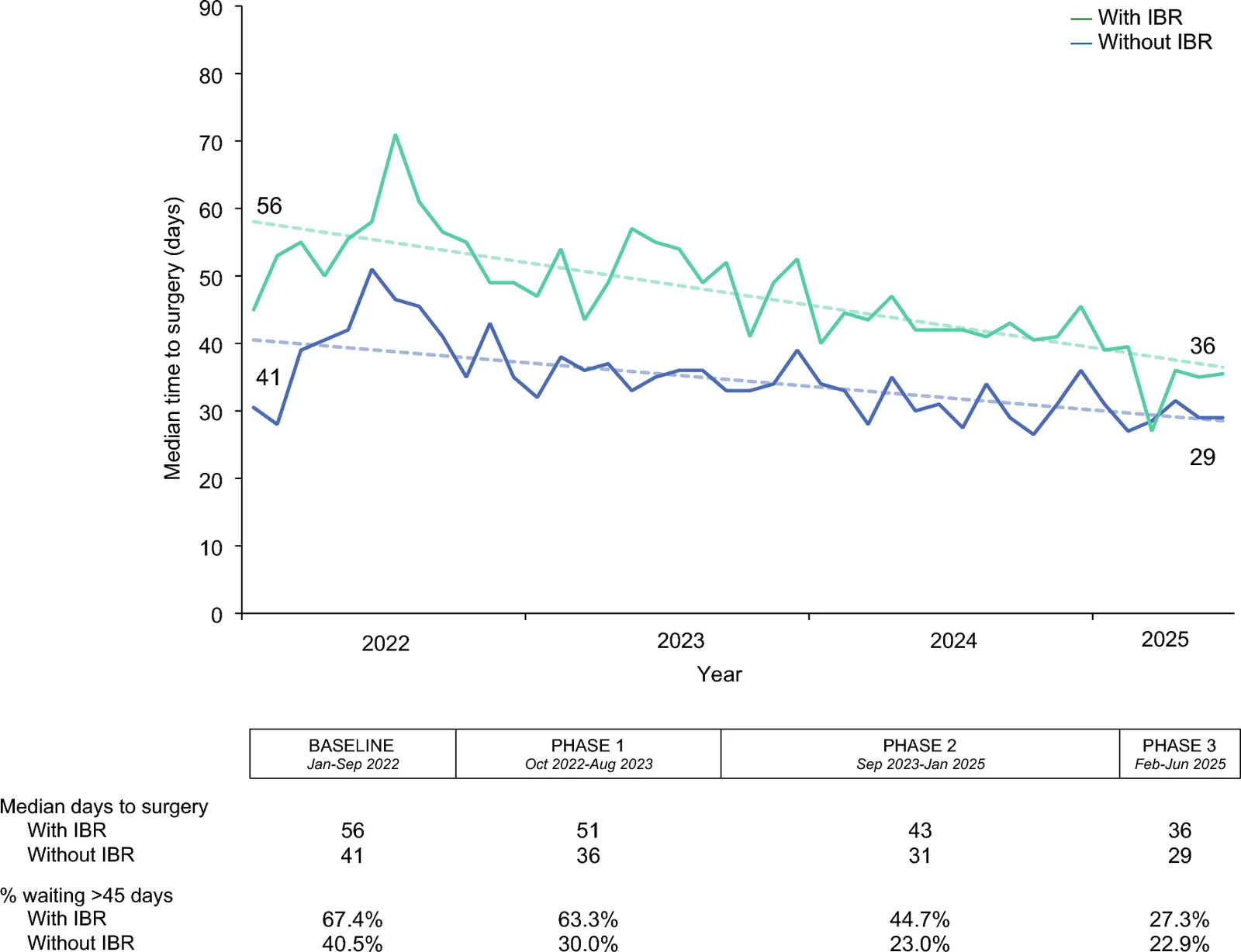
Median days to initial treatment for newly diagnosed breast cancer patients at a single institution from January 2022 to June 2025

Phase-to-phase analysis revealed a meaningful divergence in improvement trajectory between the two cohorts after Phases 1 and 2. Phase 1 only had a statistically significant impact on the surgery without IBR cohort. However, while the surgery without IBR cohort plateaued following Phase 2, the surgery with IBR cohort continued to demonstrate statistically significant improvement through Phase 3. The complete phase-to-phase analysis of median TTT and percentage of patients waiting over 45 days with associated Bonferroni-adjusted *P* values for both cohorts are presented in Table 2. Phase-by-phase reduction in median TTT for both cohorts across the study period is illustrated in Fig. 1.

## Discussion

This study demonstrated that a targeted, stepwise multidisciplinary QI initiative reduced TTT for patients undergoing upfront breast cancer surgery with IBR by three weeks. The median TTT in the IBR cohort declined from 56 days at baseline to 36 days following full program implementation, representing a 35.7% improvement. The communication protocol (Phase 2) and targeted OR alignment between breast surgery and plastic surgery (Phase 3) were the most impactful interventions for the IBR group, reducing TTT by one week each.

Delays associated with immediate breast reconstruction are well-characterized in the literature. Unlike breast surgery alone, IBR requires coordinating schedules of two independent surgical teams, each with distinct preoperative planning requirements, separate operative schedules, and competing OR block time.^8^ Late referrals to plastic surgery also delay time to surgery.^8,10,11^ Our standardized communication protocol was designed primarily to expedite OR scheduling rather than initial plastic surgery appointments. This centralized model eliminated the fragmented, ad hoc communication between services that had previously contributed to scheduling delays and ensured that IBR cases were actively tracked from patient’s entry into our multidisciplinary clinic. Recently, the PROMPT Quality Collaborative identified the most effective QI interventions directed at breast centers to reduce biopsy-to-treatment time, and found that increasing OR block time for combined breast and plastic surgery cases reduced TTT by 39.5 days.^12^ McKevitt and colleagues describe a swing room scheduling strategy where a surgical oncologist and plastic surgeon move between two concurrently running ORs to complete several combined mastectomy cases in one day.^13^ In our study, alignment of OR time, which also includes utilization of two rooms by the breast and plastics team, showed a meaningful reduction in time to breast surgery with IBR. This tailored, volume-based approach differs from the fixed block scheduling or swing room models described in the literature, in which OR time is allocated in advance without reference to actual institutional case volume. By grounding our allocation in observed demand, we were able to match supply to need at the level of individual surgeon pairs, minimizing unused block time while maximizing availability for IBR cases. On average at our institution, each breast surgeon has two to five dedicated IBR days per month, with case volume varying by breast surgeon–plastic surgeon pairing.

Our institution has a longstanding commitment to reducing TTT for newly diagnosed cancer patients through continuous quality improvement, having previously demonstrated meaningful reductions in TTT following implementation of multidisciplinary coordination and value-stream mapping across its cancer programs.^14^ Building on this institutional precedent and literature that supports the effectiveness of optimized scheduling, our breast surgical oncology and plastic and reconstructive surgery teams mapped the current process from time from clinic visit to surgery and identified root causes for delays in care. Following this, the team collaboratively identified countermeasures and initiated a targeted initiative specifically designed to address the structural delays associated with IBR scheduling. OR alignment implemented in Phase 3 was one of the most effective interventions for the IBR cohort. This finding also emphasizes that the root cause of IBR delay is fundamentally a scheduling problem. Visual management and communication protocols can optimize information flow and reduce administrative delays, but they cannot resolve the underlying structural bottleneck of misaligned operative availability. Tailored OR block alignment directly addresses this root cause by ensuring that combined breast surgery and plastic surgery cases can be booked into coordinated OR time from the outset based on each surgeon’s IBR case volume.

Beyond median TTT, the proportion of patients waiting longer than 45 days is an internal threshold we used to identify outliers experiencing delays in care. At baseline, 67.4% of IBR patients and 40.5% of non-IBR patients exceeded this threshold. By Phase 3 of the intervention, the proportion of IBR patients waiting longer than 45 days declined to 27.3%, and the proportion of non-IBR patients declined to 22.9%. This demonstrates that, in addition to shifting the median, this QI initiative was effective in reducing the longest wait times across the study cohort. Our program continues to identify opportunities to further reduce the number of patients with delays over 45 days.

Quality improvement initiatives in complex multidisciplinary settings carry the inherent risk of zero-sum tradeoffs where improvements achieved for one patient population may come at the expense of another if shared resources are finite. This concern is particularly salient in the context of OR scheduling, where gains in block time for combined IBR cases could potentially displace access for nonreconstructive patients. The inclusion of a non-IBR balancing metric served as a critical safeguard and is a strength of this study design. Our data demonstrated that improvements to IBR TTT did not come at the expense of non-IBR TTT. Median TTT and proportion waiting longer than 45 days for the surgery without IBR cohort also improved over the study period—declining from 41 to 29 days and from 40.8 to 22.7%, respectively. Simultaneous improvement in both cohorts suggests that OR alignment achieved efficiency gains that benefited the system as a whole.

Our findings have direct clinical implications for breast programs seeking to improve IBR TTT. The three-phase initiative described here is notable for its reproducibility and scalability: weekly multidisciplinary huddles, standardized communication protocols, and coordinated OR block scheduling are low-cost and operationally straightforward interventions that do not require additional financial investment, personnel, or significant infrastructure changes. The sequential, phased approach also offers a practical implementation roadmap for interventions that build on each other over time. This iterative model aligns with established continuous improvement principles and may be particularly well-suited to academic medical centers where breast surgery and plastic surgery operate as separate departments with independent scheduling systems. Taken alongside our institution's prior experience demonstrating significant TTT reduction through multidisciplinary coordination and value-stream mapping^14^ and work showing that once patients enter our care pathway treatment timelines are highly efficient regardless of diagnosis location,^15^ these results reflect our latest quality improvement efforts across the breast care pathway.

Several limitations of this study should be acknowledged. First, this was a single-institution retrospective study, which limits generalizability and introduces the potential for unmeasured confounders including changes in case variety, provider volume, or institutional capacity over the study period. Second, the study period partially overlaps with the post-COVID recovery era, during which operative backlogs and scheduling disruptions may have influenced baseline TTT independent of the QI intervention. Third, patient-reported outcomes, including satisfaction with the timing of surgery and psychosocial measures related to treatment delays, were not captured and represent an important dimension of care quality that TTT metrics alone cannot fully characterize. Fourth, we did not assess the downstream impact of TTT improvements on adjuvant therapy initiation, which is an important oncologic endpoint given the known association between IBR and delays in adjuvant treatment.^16,17^ Fifth, while the non-IBR balancing metric provides reassurance against zero-sum tradeoffs in surgical access, it does not capture potential effects on outpatient clinic capacity, preoperative workup timelines, or other resource domains.

Future directions for this work include a formal disparity analysis examining TTT by insurance status and other socioeconomic factors across phases of the QI initiative. Assessment of adjuvant therapy timing as a downstream outcome, patient satisfaction and quality-of-life data, and a formal cost analysis of the initiative would further characterize its value. Multi-institutional validation of the OR alignment model would strengthen the generalizability of our findings and facilitate broader adoption across academic breast programs.

## Conclusions

Implementation of a targeted multidisciplinary QI initiative meaningfully reduced TTT for breast cancer patients undergoing upfront surgery with IBR, with median TTT declining from 56 to 36 days over the study period. OR alignment was a pivotal intervention, driving continued improvement in the IBR cohort. Simultaneous improvement in the non-IBR balancing measure confirms that these gains were achieved without displacing care for non-reconstructive patients. This stepwise, reproducible model offers a practical framework for academic breast programs seeking to improve the timeliness of IBR without compromising broader surgical access.
